# Levels of Cholesterol in Small LDL Particles Predict Atherosclerosis Progression and Incident CHD in the HDL-Atherosclerosis Treatment Study (HATS)

**DOI:** 10.1371/journal.pone.0056782

**Published:** 2013-02-27

**Authors:** Paul T. Williams, Xue-Qiao Zhao, Santica M. Marcovina, B. Greg Brown, Ronald M. Krauss

**Affiliations:** 1 Life Sciences Division, Lawrence Berkeley Laboratory, Berkeley, California, United States of America; 2 Department of Medicine, Division of Cardiology, University of Washington, Seattle, Washington, United States of America; 3 Department of Medicine, Northwest Lipid Research Laboratories, University of Washington, Seattle, Washington, United States of America; 4 Children's Hospital Oakland Research Institute, Oakland, California, United States of America; University of Buenos Aires, Cardiovascular Pathophysiology Institute, Argentina

## Abstract

**Objective:**

Test whether angiographically-documented changes in percent stenosis and clinical endpoints (coronary-related deaths, myocardial infarctions, stroke, revascularization for worsening ischemia) in the HDL-Atherosclerosis Treatment Study (HATS) were attributable to specific LDL-subclasses.

**Methods:**

Gradient gel electrophoresis of on-study LDL-subclass cholesterol concentrations were measured in 32 placebo, 33 simvastatin-niacin, 38 antioxidant, and 39 simvastatin-niacin & antioxidant treated participants. The prespecified primary end point was the mean change per patient from the initial arteriogram to the final arteriogram in the percent stenosis caused by the most severe lesion in each of the nine proximal coronary segments.

**Results:**

The change in the percent stenosis of the most severe proximal lesions increased in association with higher concentrations of the small LDL subfractions LDL-IIIb (24.2–24.6 nm) and LDL-IVa (23.3–24.1 nm) before (both P = 0.002) and after (P = 0.01 and P = 0.03 respectively) adjustment for treatment group and on-study HDL-cholesterol, LDL-cholesterol, and triglyceride concentrations. The associations appeared specific to lesions with <30% baseline stenosis. When adjusted for age, sex, baseline BMI and cigarette use, the odds for primary clinical endpoints (death from coronary causes, nonfatal myocardial infarction, stroke, or revascularization for worsening ischemia) were significantly greater in subjects with higher on-study LDL-IIIb levels both before (P = 0.01) and after (P = 0.03) adjustment for treatment group and the standard lipid values.

**Conclusions:**

Plasma LDL-IIIb cholesterol concentrations were related to changes in coronary artery stenosis and cardiovascular events in patients with coronary artery disease and low HDL-cholesterol.

**Trial Registration:**

ClinicalTrials.gov NCT00000553

## Introduction

The HDL-Atherosclerosis Treatment Study (HATS) was a double-blind randomized controlled clinical trial of simvastatin plus niacin and/or a mixture of antioxidants in 160 patients selected for clinical coronary disease with at least 3 stenoses of at least 30 percent of the luminal diameter or 1 stenosis of at least 50 percent, and low high-density lipoprotein (HDL)-cholesterol [1]. The study demonstrated that simvastatin plus niacin treatment reduced the rate of progression of angiographically-documented coronary artery stenoses relative to placebo treatment, and reduced the frequency of clinical endpoints (3 percent) relative to placebo (24 percent). Mean changes in the severity of proximal stenosis during the trial were significantly correlated with on-study levels of low-density lipoprotein (LDL)-cholesterol (r = 0.22, p = 0.008), as well as with other lipoprotein measurements [1].

LDL is known to comprise multiple subclasses differing in size and density and in their relationships with cardiovascular disease risk [2]–[4]. The technique of non-denaturing gradient gel electrophoresis was first used to identify seven distinct size subclasses of LDL particles [2], and has subsequently been adapted to provide a measure of the plasma concentration of cholesterol in each of these subclasses [5]. In the present report, this method was employed in HATS to assess whether coronary atherosclerosis progression and clinical endpoints in HATS could be attributed to on-study plasma levels of specific LDL subclasses.

## Methods

### Clinical procedures

The study protocol has been described in detail elsewhere [1] and is therefore summarized only briefly. Patients (men <63 yr and women <70 yr) were recruited on the basis of having had clinical coronary disease defined as previous myocardial infarction, coronary interventions, or confirmed angina with required percent stenosis, and low HDL cholesterol (≤35 mg/dl for men and ≤40 mg/dl for women). Plasma LDL-cholesterol was restricted to ≤145 mg/dl and plasma triglycerides to ≤400 mg/dl. Enrollment occurred between 1995 and 1997 in the United States (134 patients) and Canada (26 patients). Treatment assignment was random and double-blinded. The study was approved by the University of Washington institutional review committee and all patients signed informed consent as approved by the human use committee.

Patients assigned to simvastatin-niacin treatment (with or without antioxidant vitamins) began receiving 10 mg/d simvastatin (Zocor, Merck, West Point, Pa.) if their screening LDL-cholesterol was less than 110 mg/dl and 20 mg/d if over 110 mg/dl. The dose was increased by an additional 10 mg/dl if the LDL-cholesterol did not consistently remain below 110 mg/dl and was reduced by 10 mg/d if their LDL-cholesterol fell below 40 mg/dl. Those in either the placebo or the antioxidant treatment arm received simvastatin if their LDL-cholesterol exceeded 140 mg/dl. Slow release niacin increased from two daily doses of 250 mg (Slo-Niacin, Upsher–Smith, Minneapolis) to two daily doses of 1000 mg during the first month of the trial. Crystalline (Niacor, Upsher–Smith) replaced slow-release niacin if HDL-cholesterol levels were not increased by at least 5, 8, and 10 mg/dl after 3, 8 and 12 months, respectively. Two 50 mg tablets of active niacin were given as placebo to elicit a flushing response. The antioxidant consisting of 800 IU of vitamin E (as d-alpha-tocopherol), 1000 mg of vitamin C, 25 mg of natural beta carotene, and 100 g of selenium was administered twice daily as gel capsules that were indistinguishable from the placebo. All patients were encouraged to enter an exercise program, increase monounsaturated fat intake, and stop smoking, and were counseled for weight loss.

### Laboratory measurements

Plasma LDL-cholesterol, HDL-cholesterol, and triglyceride concentrations were measured by Northwest Lipid Research Laboratories by procedures that were in accord with the NHLBI/CDC Lipid Standardization Program [1]. In addition, LDL-cholesterol was determined at the 12 month time point by a vertical rotor ultracentrifugation procedure that excludes the contribution of intermediate density lipoproteins and Lp(a) to the measurement as defined by the NHLBI/CDC program [6].

LDL subclasses were analyzed using non-denaturing gradient gel electrophoresis (GGE) of fasting samples taken at baseline and after 24 months. Aliquots of 3.0 mL of whole plasma were mixed 1:1 with a sampling buffer of 20% sucrose and 0.25% bromophenol blue. Electrophoresis of samples and size calibration standards was performed using 2%–14% polyacrylamide gradients at 150 V for 3 hours following a 15-minute pre-run at 75 V as described previously [2]. Gels were stained with 0.07% Sudan black for 1 hr and stored in a 0.81% acetic acid, 4% methanol solution until they were scanned by computer-assisted densitometry for determination of areas of LDL-IVb (22.0–23.2 nm), LDL-IVa (23.3–24.1 nm), LDL-IIIb (24.2–24.6 nm), LDL-IIIa (24.7–25.5 nm), LDL-IIb (25.6–26.4 nm), LDL-IIa (26.5–27.1 nm), and LDL-I (27.2–28.5 nm). Since the absorbance profiles of LDL particles stained with Sudan Black have been shown to reflect the cholesterol distribution among LDL subclasses [5], the cholesterol concentrations of the subclasses were determined by multiplying percent of the total stained LDL for each subclass by the cholesterol measured in the ultracentrifugally isolated LDL fractions. Although this measurement was not available at the 24 month time point, the use of the 12 month value was taken to be representative of the overall on-study LDL-cholesterol level [1].

Arteriography was performed and interpreted without knowledge of the patient's treatment assignment. At baseline and follow-up, eight views of the left and right coronary arteries were filmed after sublingual administration of 0.2 to 0.4 mg nitroglycerin [1]. The locations of all lesions that caused ≥15% stenosis of the luminal diameter were mapped. Baseline and follow-up images were compared side-by-side to measure the minimal luminal diameter (Diameter_minimum_) and nearby normal diameters (Diameter_normal_) in millimeters using the catheter for calibration. Stenosis was expressed as a percentage (i.e., 100^*^(Diameter_normal_ -Diameter_minimum_)/(Diameter_normal_)). The prespecified primary end point was the mean change per patient from the initial arteriogram to the final arteriogram in the percent stenosis caused by the most severe lesion in each of the nine proximal coronary segments. Secondary end points included the mean change in percent stenosis in lesions of varying degrees of severity. Results are also presented for all stenotic lesions measured in each patient. Prespecified primary clinical endpoints were death from coronary causes, nonfatal myocardial infarction, stroke, or revascularization for worsening ischemia.

Statistics. Analysis of variance was used to assess the significance of the mean differences between groups, standard least-squares regression analyses were used to assess the association between changes in percent stenosis and on-study lipoprotein concentrations, and logistic regression analyses were used to test for significant associations between the odds for primary clinical endpoints and LDL-peak diameter and LDL-subclass concentrations. Baseline age, sex, BMI, current smoking status, and standard clinical lipid measurements (on study HDL-cholesterol, LDL-cholesterol, and triglyceride concentrations) were included as covariates where indicated. All hypotheses were two-sided.

## Results

One hundred forty six of the original 160 patients enrolled into the study completed the angiographic protocol [1]. One subject assigned to antioxidants, one assigned to simvastatin-niacin antioxidants, and two subjects assigned to placebo were missing either gradient gel (2 subjects) or vertical spin LDL-cholesterol (2 subjects), leaving 32 placebo treated, 33 simvastatin-niacin, 38 antioxidant, and 39 simvastatin-niacin & antioxidant treated participants for study. Table 1 presents the characteristics of this subset, which differ little from those of the complete sample as presented in the initial report [1].

**Table 1:** Baseline characteristics of study participants.

	Placebo	Simvastatin & Niacin	Antioxidant vitamins	Simvastatin, Niacin & Anitoxidant vitamins
Males (N)/Females (N)	30/2	28/5	34/4	33/6
Age	52.9±7.7	52.6±9.4	53.8±8.3	54.9±7.5
BMI (kg/m^2^)	29.7±3.9	29.3±4.0	28.7±3.4	29.9±4.9
Current Smokers (%)	18.8	24.2	26.3	23.1
LDL-peak (nm)	25.53±0.13	25.74±0.13	25.51±0.12	25.68±0.12
LDL-I (mmol/L)	0.44±0.05	0.50±0.05	0.37±0.05	0.44±0.05
LDL-IIa (mmol/L)	0.46±0.06	0.54±0.06	0.37±0.05	0.47±0.05
LDL-IIb (mmol/L)	0.73±0.06	0.74±0.06	0.64±0.06	0.71±0.06
LDL-IIIa (mmol/L)	0.58±0.06	0.57±0.05	0.63±0.05	0.59±0.05
LDL-IIIb (mmol/L)	0.18±0.02	0.13±0.02	0.15±0.02	0.15±0.02
LDL-IVa (mmol/L)	0.12±0.01	0.12±0.01	0.11±0.01	0.11±0.01
LDL-IVb (mmol/L)	0.11±0.01	0.11±0.01	0.11±0.01	0.11±0.01
LDL-C (mmol/L)	3.28±0.13	3.38±0.13	3.06±0.12	3.20±0.12
HDL-C (mmol/L)	0.82±0.02	0.82±0.02	0.83±0.02	0.78±0.02
Triglycerides (mmol/L)	2.22±0.20	2.27±0.20	2.38±0.19	2.66±0.18

Age and BMI are displayed as mean±SD, lipoproteins as means±SE

Table 2 shows that during the study, those assigned to simvastatin-niacin treatment significantly increased their LDL peak diameter and plasma HDL-cholesterol concentrations, and significantly reduced their mean plasma concentrations of LDL-cholesterol, triglycerides, and all LDL subclasses except LDL-IIIb relative to placebo assignment. On-study LDL-cholesterol levels in LDL-IIb, LDL-IIIa, LDL-IIIb, LDL-IVa and LDL-IVb were on average significantly lower in the simvastatin-niacin treatment group than placebo, as were total LDL-cholesterol and triglyceride concentrations, while HDL-C levels were significantly higher than those on placebo. Antioxidant treatment alone significantly reduced LDL-peak diameter and increased LDL-IIIb cholesterol relative to placebo. The combination of simvastatin-niacin and antioxidants significantly lowered triglycerides, LDL-cholesterol, LDL-I, LDL-IIa, LDL-IIb, and LDL-IIIa, and increased HDL-cholesterol, relative to placebo.

**Table 2:** On-study and change from baseline plasma lipoprotein subfraction concentrations (95% confidence interval) by treatment assignment.

Adjustment	Placebo	Simvastatin & Niacin	Antioxidant vitamins	Simvastatin, Niacin & Antioxidant vitamins	Anova significance for group differences
LDL-peak					
On study	25.82	26.40	25.48	26.04	
	(25.57, 26.08)	(26.15, 26.65)	(25.25, 25.71)	(25.81, 26.27)	
		*P = 0.002*	*P = 0.06*	*P = 0.21*	*P = 7.2×10^−6^*
Change	0.29	0.66	−0.03	0.36	
	(0.05, 0.53)	(0.42, 0.90)	(−0.25, 0.19)	(0.14, 0.58)	
		*P = 0.04*	*P = 0.05*	*P = 0.69*	*P = 0.0008*
LDL-I					
On study	0.48	0.40	0.40	0.35	
	(0.41, 0.54)	(0.34, 0.46)	(0.34, 0.46)	(0.29, 0.41)	
		*P = 0.09*	*P = 0.08*	*P = 0.005*	*P = 0.04*
Change	0.03	−0.10	0.04	−0.09	
	(−0.05, 0.12)	(−0.18, −0.02)	(−0.04, 0.11)	(−0.17, −0.01)	
		*P = 0.03*	*P = 0.98*	*P = 0.03*	*P = 0.02*
LDL-IIa					
On study	0.52	0.45	0.41	0.34	
	(0.44, 0.60)	(0.37, 0.53)	(0.34, 0.49)	(0.27, 0.42)	
		*P = 0.19*	*P = 0.05*	*P = 0.001*	*P = 0.01*
Change	0.06	−0.09	0.05	−0.13	
	(−0.04, 0.15)	(−0.19, 0.00)	(−0.04, 0.14)	(−0.21, −0.04)	
		*P = 0.03*	*P = 0.89*	*P = 0.008*	*P = 0.009*
LDL-IIb					
On study	0.78	0.52	0.69	0.51	
	(0.67, 0.88)	(0.41, 0.62)	(0.59, 0.79)	(0.41, 0.61)	
		*P = 0.0009*	*P = 0.25*	*P = 0.0004*	*P = 0.0005*
Change	0.05	−0.22	0.05	−0.20	
	(−0.07, 0.16)	(−0.33, −0.11)	(−0.05, 0.15)	(−0.30, −0.10)	
		*P = 0.001*	*P = 0.98*	*P = 0.002*	*P = 0.0001*
LDL-IIIa					
On study	0.52	0.28	0.72	0.38	
	(0.42, 0.62)	(0.19, 0.38)	(0.62, 0.81)	(0.29, 0.47)	
		*P = 0.001*	*P = 0.006*	*P = 0.04*	*P = 1.2×10^−8^*
Change	−0.05	−0.29	0.09	−0.21	
	(−0.16, 0.05)	(−0.39, −0.19)	(−0.01, 0.19)	(−0.30, −0.11)	
		*P = 0.002*	*P = 0.06*	*P = 0.04*	*P = 2.0×10^−5^*
LDL-IIIb					
On study	0.16	0.08	0.19	0.10	
	(0.12, 0.19)	(0.05, 0.12)	(0.15, 0.22)	(0.06, 0.13)	
		*P = 0.006*	*P = 0.23*	*P = 0.02*	*P = 0.0001*
Change	−0.03	−0.05	0.03	−0.05	
	(−0.07, 0.01)	(−0.09, −0.01)	(0.00, 0.07)	(−0.09, −0.02)	
		*P = 0.44*	*P = 0.03*	*P = 0.34*	*P = 0.006*
LDL-IVa					
On study	0.13	0.10	0.13	0.11	
	(0.11, 0.14)	(0.08, 0.11)	(0.12, 0.15)	(0.09, 0.12)	
		*P = 0.01*	*P = 0.78*	*P = 0.05*	**P = 0.007**
Change	0.01	−0.03	0.02	−0.01	
	(−0.02, 0.03)	(−0.05, 0.00)	(0.00, 0.04)	(−0.03, 0.01)	
		*P = 0.05*	*P = 0.42*	**P = 0.31**	*P = 0.03*
LDL-IVb					
On study	0.12	0.10	0.12	0.11	
	(0.11, 0.13)	(0.08, 0.11)	(0.11, 0.13)	(0.10, 0.12)	
		*P = 0.007*	*P = 0.99*	*P = 0.34*	*P = 0.02*
Change	0.01	−0.01	0.02	0.00	
	(0.00, 0.03)	(−0.03, 0.00)	(0.00, 0.03)	(−0.02, 0.02)	
		*P = 0.02*	*P = 0.80*	*P = 0.29*	*P = 0.05*
LDL-C					
On study	2.99	1.92	2.95	2.04	
	(2.80, 3.18)	(1.73, 2.11)	(2.78, 3.13)	(1.87, 2.21)	
		*P = 3.3×10^−15^*	*P = 0.77*	*P = 3.9×10^−13^*	*P<10^−15^*
Change	−0.29	−1.46	−0.10	−1.17	
	(−0.49, −0.10)	(−1.65, −1.27)	(−0.29, 0.08)	(−1.34, −0.99)	
		*P<10^−15^*	*P = 0.17*	*P = 1.6×10^−9^*	*P<10^−15^*
HDL-C					
On study	0.89	1.03	0.85	0.93	
	(0.84, 0.94)	(0.98, 1.08)	(0.81, 0.90)	(0.88, 0.98)	
		**P = 0.0003**	*P = 0.35*	*P = 0.28*	*P = 0.0001*
Change	0.07	0.22	0.03	0.15	
	(0.03, 0.11)	(0.18, 0.26)	(−0.01, 0.06)	(0.11, 0.18)	
		*P = 2.9×10^−7^*	**P = 0.13**	*P = 0.005*	*P = 4.9×10^−10^*
Triglycerides					
On study	2.13	1.40	2.73	1.85	
	(1.67, 2.59)	(0.95, 1.85)	(2.30, 3.15)	(1.43, 2.26)	
		*P = 0.03*	*P = 0.06*	*P = 0.37*	*P = 0.0005*
Change	−0.09	−0.87	0.34	−0.82	
	(−0.48, 0.30)	(−1.25, −0.49)	(−0.01, 0.70)	(−1.17, −0.47)	
		*P = 0.006*	*P = 0.11*	*P = 0.007*	*P = 4.9×10^−6^*

Table 3 displays the relationships of the on-study LDL-subclass cholesterol concentrations to average change in percent stenosis. Analyses are presented for the most severe lesions at baseline and separately for lesions with >50%, 30–49%, or <30% stenosis at baseline. The percent stenosis of the most severe proximal lesions increased in association with higher on-study concentrations of LDL-IIIb, LDL-IVa and LDL-IVb, and smaller on-study LDL-peak diameter, without adjustment for treatment group assignment, and increased significantly with higher on-study concentrations of LDL-IIIb and LDL-IVa when further adjusted for treatment assignment, and both treatment assignment and on study HDL-cholesterol, LDL-cholesterol, and triglyceride concentrations. The associations between the changes in percent stenosis and the LDL subclasses appeared to be most specific to those lesions at baseline with <30% stenosis (LDL-IIIb and LDL-IVa, and LDL-peak diameter).

**Table 3:** Regression slope (95% confidence interval) for three-year changes in percent stenosis per nm increase in on-study LDL-peak diameter and per mmol/L increase LDL-cholesterol subfraction concentrations.

Adjustment	All	Lesions with ≥50% stenosis at baseline	Lesions with 30–49% stenosis at baseline	Lesions with 0–29% stenosis at baseline
LDL-peak				
***Standard covariates***	−0.98	−0.44	−1.22	−1.51
	(−1.84, −0.12)	(−3.1, 2.22)	(−2.85, 0.42)	(−2.77, −0.25)
	*P = 0.03*	*P = 0.75*	*P = 0.15*	*P = 0.02*
Standard covariates & group	−0.58	−0.18	−1.01	−1.00
	(−1.48, 0.33)	(−3.03, 2.67)	(−2.79, 0.77)	(−2.39, 0.39)
	*P = 0.22*	*P = 0.90*	*P = 0.27*	*P = 0.16*
Standard covariates, group & lipids	−0.77	−1.37	−1.49	−1.35
	(−1.87, 0.34)	(−4.67, 1.94)	(−3.66, 0.67)	(−3.04, 0.34)
	*P = 0.18*	*P = 0.42*	*P = 0.18*	*P = 0.12*
LDL-I				
***Standard covariates***	−0.06	3.08	−0.26	−2.02
	(−3.81, 3.69)	(−7.91, 14.06)	(−7.29, 6.77)	(−7.58, 3.54)
	*P = 0.97*	*P = 0.58*	*P = 0.94*	*P = 0.48*
Standard covariates & group	−1.57	2.12	−1.25	−3.49
	(−5.23, 2.09)	(−9.08, 13.32)	(−8.48, 5.98)	(−9.02, 2.05)
	*P = 0.40*	*P = 0.71*	*P = 0.73*	*P = 0.22*
Standard covariates, group & lipids	−1.74	−1.51	−1.34	−6.09
	(−6.36, 2.87)	(−15.49, 12.47)	(−10.4, 7.71)	(−13.11, 0.93)
	*P = 0.46*	*P = 0.83*	*P = 0.77*	*P = 0.09*
LDL-IIa				
***Standard covariates***	−1.37	−2.34	−2.23	−0.52
	(−4.32, 1.57)	(−11.57, 6.88)	(−7.71, 3.26)	(−4.94, 3.90)
	*P = 0.36*	*P = 0.62*	*P = 0.43*	*P = 0.82*
Standard covariates & group	−2.56	−1.50	−3.02	−1.63
	(−5.44, 0.33)	(−11.06, 8.06)	(−8.71, 2.67)	(−6.11, 2.85)
	*P = 0.09*	*P = 0.76*	*P = 0.30*	*P = 0.48*
Standard covariates, group & lipids	−2.92	−5.18	−3.78	−2.82
	(−6.38, 0.53)	(−16.69, 6.32)	(−10.62, 3.05)	(−8.16, 2.52)
	*P = 0.10*	*P = 0.38*	*P = 0.28*	*P = 0.30*
LDL-IIb				
***Standard covariates***	0.61	2.84	−1.31	1.72
	(−1.56, 2.77)	(−3.55, 9.24)	(−5.34, 2.73)	(−1.52, 4.96)
	*P = 0.58*	*P = 0.39*	*P = 0.53*	*P = 0.30*
Standard covariates & group	−0.98	2.78	−2.66	−0.06
	(−3.17, 1.21)	(−4.14, 9.7)	(−6.98, 1.66)	(−3.48, 3.35)
	*P = 0.38*	*P = 0.43*	*P = 0.23*	*P = 0.97*
Standard covariates, group & lipids	−0.77	2.26	−3.13	−0.60
	(−3.34, 1.81)	(−5.53, 10.04)	(−8.20, 1.94)	(−4.60, 3.40)
	*P = 0.56*	*P = 0.57*	*P = 0.23*	*P = 0.77*
LDL-IIIa				
***Standard covariates***	1.77	0.50	1.29	2.89
	(−0.34, 3.88)	(−5.48, 6.47)	(−2.84, 5.42)	(−0.20, 5.99)
	*P = 0.10*	*P = 0.87*	*P = 0.54*	*P = 0.07*
Standard covariates & group	0.51	0.39	0.45	1.11
	(−1.83, 2.85)	(−7.00, 7.78)	(−4.40, 5.30)	(−2.49, 4.72)
	*P = 0.67*	*P = 0.92*	*P = 0.86*	*P = 0.55*
Standard covariates, group & lipids	0.85	1.19	0.90	0.73
	(−1.60, 3.30)	(−6.58, 8.96)	(−4.35, 6.15)	(−3.07, 4.52)
	*P = 0.50*	*P = 0.76*	*P = 0.74*	*P = 0.71*
LDL-IIIb				
***Standard covariates***	9.67	−1.41	9.50	23.29
	(3.80, 15.55)	(−17.88, 15.05)	(−1.91, 20.92)	(15.27, 31.3)
	*P = 0.002*	*P = 0.87*	*P = 0.11*	*P = 1.2×10^−8^*
Standard covariates & group	7.20	−2.67	8.27	21.78
	(1.04, 13.35)	(−20.15, 14.81)	(−3.92, 20.47)	(13.05, 30.52)
	*P = 0.02*	*P = 0.77*	*P = 0.19*	*P = 10^−6^*
Standard covariates, group & lipids	8.15	−0.10	9.76	22.60
	(1.73, 14.56)	(−18.48, 18.29)	(−3.30, 22.82)	(13.44, 31.75)
	*P = 0.01*	*P = 0.99*	*P = 0.15*	*P = 10^−6^*
LDL-IVa				
***Standard covariates***	20.51	3.12	30.09	32.08
	(6.91, 34.12)	(−39.62, 45.87)	(3.68, 56.51)	(11.92, 52.24)
	*P = 0.002*	P = 0.89	*P = 0.03*	*P = 0.002*
Standard covariates & group	14.62	−1.40	26.78	25.58
	(0.92, 28.32)	(−45.27, 42.46)	(−0.61, 54.18)	(4.7, 46.46)
	*P = 0.04*	*P = 0.95*	*P = 0.06*	*P = 0.02*
Standard covariates, group & lipids	15.89	2.20	30.17	25.08
	(1.68, 30.11)	(−43.06, 47.47)	(1.48, 58.85)	(3.19, 46.98)
	*P = 0.03*	*P = 0.92*	*P = 0.04*	*P = 0.03*
LDL-IVb				
***Standard covariates***	19.05	13.07	33.73	28.15
	(0.87, 37.22)	(−50.85, 76.99)	(−0.82, 68.28)	(1.08, 55.23)
	P = 0.04	P = 0.69	P = 0.06	P = 0.04
Standard covariates & group	11.24	6.13	28.20	21.07
	(−6.85, 29.32)	(−59.49, 71.75)	(−8.04, 64.43)	(−6.60, 48.74)
	*P = 0.23*	*P = 0.86*	*P = 0.13*	*P = 0.14*
Standard covariates, group & lipids	12.78	12.98	33.51	19.31
	(−6.18, 31.74)	(−55.65, 81.61)	(−5.07, 72.09)	(−9.94, 48.55)
	*P = 0.19*	*P = 0.71*	*P = 0.09*	*P = 0.20*

The regression slope (±SE) between on-study LDL-IIIb and most severe proximal lesions persisted when adjusted for on-study apo B concentrations (all: 8.30±3.31% per mmol/L, P = 0.01; <30% stenosis: 22.55±4.56, P<10^−6^), or on-study total/HDL-cholesterol (all: 8.62±3.41% per mmol/L, P = 0.01; <30% stenosis: 23.40±4.66, P<10^−6^), and in an analyses of all 7 LDL subclasses included simultaneously (all: 11.07±5.77% per mmol/L, P = 0.06; <30% stenosis: 47.92±7.55, P<10^−9^). Large LDL (LDL-I or LDL-IIa) was unrelated to change in percent stenosis with or without adjustment for group and standard lipid measurements (Table 3), and in the simultaneous analyses of all 7 LDL subfractions.

**Figure 1:**
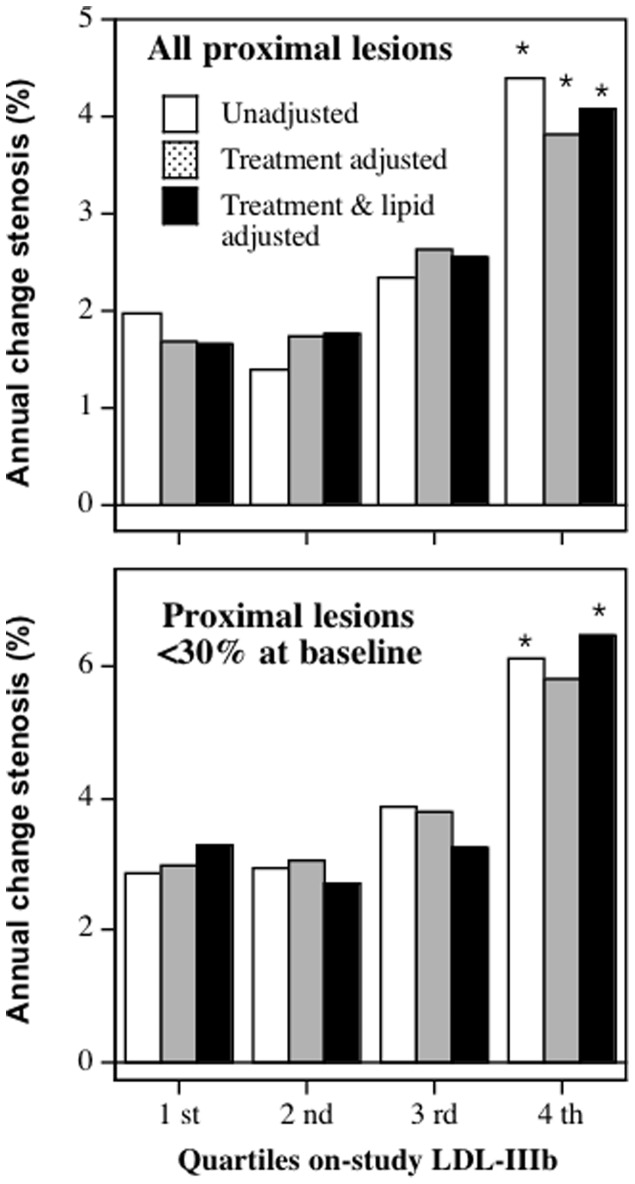
Three-year change in percent stenosis by quartiles of LDL-IIIb cholesterol. Adjusted for age, sex, BMI and smoking, and additional variables as indicated. Adjustment for lipids includes on-study HDL- and LDL-cholesterol and triglyceride concentrations. ^*^ designates significance relative to the first quartile at P<0.05.

Figure 1 presents the changes in percent stenosis in all proximal lesions and in proximal lesions with less than 30% stenosis at baseline by quartiles of on-study LDL-IIIb cholesterol concentrations. The changes in percent stenosis were adjusted to male nonsmokers of average age and BMI for the sample. Additional adjustments for treatment group assignment, and treatment group assignment plus on-study lipids (HDL-cholesterol, LDL-cholesterol, and triglyceride concentrations) were made where indicated. Consistent with the significant regression slopes of Table 3, the figure shows that those in the highest quartile of LDL-IIIb cholesterol had significantly greater increases in percent stenosis than those in the lowest quartile. Specifically, the significance of the 4^th^ vs. 1^st^ quartile differences for all proximal lesions and those with <30% stenosis were P = 0.02 and P = 0.04 without adjustment for treatment group, P = 0.03 and P = 0.06 when adjusted for treatment group, and P = 0.02 and P = 0.05 when adjusted for treatment group and on-study lipid levels, respectively.

Table 4 presents the logistic regression analyses of the primary clinical endpoint. There were 19 clinical events. When adjusted for age, sex, baseline BMI and smoking status, the proportion of individuals experiencing a primary clinical endpoint was significantly greater in patients with higher on-study LDL-IIIb cholesterol levels (P = 0.01). Higher on-study plasma LDL-IIIb levels remained significantly related to the odds for a primary clinical event when further adjusted for both treatment group and standard lipid values (P = 0.03), and when all 7 LDL subclasses were included simultaneously in the model (P = 0.01, analyses not displayed). Large LDL (LDL-I or LDL-IIa) was unrelated to incident events with or without adjustment for group and standard lipid measurements (Table 4), and in the simultaneous analyses of all 7 LDL-subfractions (P>0.65, not displayed). The significant odds ratio for primary clinical endpoints with greater LDL-IIIb persisted when adjusted for apo B concentrations (1.68 per 0.11 mmol/L, 95% CI: 1.04–2.71, P = 0.03), or total/HDL-cholesterol (1.78 per 0.11 mmol/L, 95% CI: 1.06–2.98, P = 0.03).

**Table 4:** Odds ratio (95% confidence interval) for primary disease endpoints (coronary-related deaths, myocardial infarctions, stroke, revascularization for worsening ischemia) per a one standard deviation increment in on-study LDL-peak diameter and LDL-subclass concentrations.

Additional adjustments	No additional adjustment	Additional adjustment for:	
		Group	Group and lipids
LDL-peak	0.70	0.84	0.87
	(0.41, 1.19)	(0.47, 1.47)	(0.43, 1.79)
	P = 0.19	P = 0.53	P = 0.71
LDL-I	0.85	0.80	0.85
	(0.49, 1.46)	(0.45, 1.42)	(0.42, 1.75)
	P = 0.55	P = 0.44	P = 0.67
LDL-IIa	0.76	0.76	0.84
	(0.44, 1.32)	(0.43, 1.35)	(0.41, 1.71)
	P = 0.33	P = 0.35	P = 0.63
LDL-IIb	0.83	0.73	0.81
	(0.49, 1.43)	(0.42, 1.27)	(0.42, 1.57)
	P = 0.51	P = 0.27	P = 0.54
LDL-IIIa	1.36	1.08	1.25
	(0.84, 2.22)	(0.63, 1.84)	(0.70, 2.24)
	P = 0.21	P = 0.79	P = 0.45
LDL-IIIb	1.73	1.56	1.77
	(1.12, 2.69)	(0.98, 2.47)	(1.07, 2.93)
	P = 0.01	P = 0.06	P = 0.03
LDL-IVa	1.18	1.05	1.08
	(0.74, 1.87)	(0.64, 1.71)	(0.64, 1.83)
	P = 0.48	P = 0.86	P = 0.76
LDL-IVb	1.13	0.96	1.03
	(0.67, 1.91)	(0.54, 1.74)	(0.55, 1.90)
	P = 0.66	P = 0.90	P = 0.94

Logistic regression analyses adjusted for baseline age, sex, BMI, current smoking status. Effects are per standard deviation increase in the on-study LDL-peak diameter (8.050 nm), LDL-I (0.185 mmol/L), LDL-IIa (0.234 mmol/L), LDL-IIb (0.326 mmol/L), LDL-IIIa (0.328 mmol/L), LDL-IIIb (0.113 mmol/L), LDL-IVa (0.049 mmol/L), and LDL-IVb (0.039 mmol/L).

## Discussion

Here we report that low on-study levels of cholesterol in small LDL particles (LDL-IIIb and LDL–IVa as identified by gradient gel electrophoresis) are associated with reduced rate of coronary atherosclerosis progression in patients followed prospectively in HATS, and that these relationships are independent of standard lipid levels. Moreover, on-study LDL-IIIb was independently associated with the primary clinical cardiovascular endpoint in HATS. The analyses also adjusted for treatment assignment because of concerns that simvastatin–niacin treatment might decrease coronary artery disease progression and reduce the risk for clinical events independent of its direct effects on lipoprotein levels. Without group adjustment, an association between the subclass concentrations and progression or clinical endpoints could simply be secondary to primary effects of simvastatin-niacin both lowering LDL-subclass concentrations and affecting disease progression. Otherwise stated, on-study subclass level might simply be a surrogate measure for group assignment. The retained significance of on-study LDL-IIIb and LDL-IVa when adjusted for treatment group shows that this is not the case. This approach also renders the results conservative because the adjustment also reduces the influence of antiatherogenic effects of the LDL-subclass reductions produced by the treatment.

The findings are consistent with prior evidence linking levels of cholesterol in small LDL particles to progression of coronary atherosclerosis as assessed by quantitative coronary angiography [7] and association between small LDL particles and native CAD progression following PTCA or CABG [8]. In the present study, this relationship was strongest for LDL-IIIb and IVa; in the case of the smallest LDL particles (LDL-IV), the associations were not consistent for all statistical adjustments. Although it has been suggested that specific properties of smaller vs. larger LDL particles, such as greater arterial proteoglycan binding and oxidative susceptibility, may be directly responsible for their enhanced atherogenic potential, there is insufficient evidence to assess the extent to which increased levels of these particles denote other pathologic mechanisms [3], [4]. In this regard, it is of interest that increased concentrations of small LDL particles (specifically LDL-IIIb and LDL-IVa and IVb) have been found to be selectively associated with a common single nucleotide polymorphism that increases hepatic apo B secretion and reduces LDL catabolism via upregulation of hepatic sortilin, and is linked to increased CHD risk [9], [10].

Our results are also consistent with the association of CHD with apo C-III containing LDL [11]–[13], in that apoC-III is enriched in the smallest LDL particles [14]. The apo C-III containing lipoproteins are hypothesized to promote atherosclerosis by activating circulating monocytes to adhere to vascular endothelial cells [15] and altering LDL composition to promote LDL adhesion to the subendothelial extracellular matrix [16]. ApoC-III also inhibits receptor-mediated uptake of VLDL remnant particles [17]. Large LDL containing apo C-III, produced by the intravascular lipolysis of apoC-III containing VLDL, are metabolized to form small LDL containing apo C-III [17]. Although Mendivol et al. surmised that the LDL containing apo C-III were restricted to larger particles, their conclusion was inferred indirectly from the ratio of cholesterol to apo B ratio rather than the direct density isolation of LDL-subfractions [12], as performed by Shin et al [14]. Moreover, apoCIII is enriched in triglyceride-rich lipoprotein remnants, which can overlap the upper range of the LDL size distribution.

The relationship of small LDL cholesterol to angiographic progression of coronary artery disease was strongest for proximal lesions with <30% luminal narrowing. Simvastatin-niacin treatment was previously reported to produce the most statistically significant difference on this category of lesions [1].

One limitation of the current study is the absence of 1.019 <d<1.063 LDL-cholesterol measurements at 24 months. Although 24-month Friedewald calculations of LDL-cholesterol were available [18], these are both indirect and include IDL-cholesterol in addition to LDL-cholesterol. The calculated on-study LDL-IIIb cholesterol concentrations based on Friedewald values at 24 months did achieve statistical significance for relation to the change in percent stenosis for proximal vessels with <30% stenosis at baseline (P<0.0001 unadjusted for treatment assignment, P = 0.002 adjusted for treatment assignment, and P = 0.003 adjusted for both treatment assignment and other lipoproteins), but not for relation to change of all proximal vessels (P = 0.002, P = 0.15, and P = 0.13, respectively), or to primary clinical endpoints (P = 0.03, P = 0.16, P = 0.10, respectively, analyses not displayed). This suggests to us that the inaccuracies in applying the Friedewald estimates to the LDL-particle distribution at 24 months were greater than those created by using the direct measurement of LDL-cholesterol at 12 months despite the separation in time of these measurements. The decision to use the 12 month vertical spin LDL-cholesterol was made a priori by one of us (RMK) independent of the analyses. In fact, the LDL-IIIb cholesterol concentrations calculated using the 24-month Friedewald LDL-cholesterol values differed little from those calculated from LDL-cholesterol measured directly at 12 months, just enough to fall short of 5% significance for all vessels and clinical endpoints. Other than providing a more accurate assessment of the on-study LDL-subclass concentrations, we can think of no plausible explanation for greater significance using LDL-cholesterol measured directly at 12 months than the 24-month Friedewald estimation. The earlier reports from this study also assume measurements taken at various time points during the intervention were representative of on-study exposures [1].

The analyses presented in the current paper were not designed to test the efficacy of simvastatin-niacin per se, nor whether treatment benefits are dependent upon LDL-cholesterol levels, but rather to identify associations of on-study LDL-subfractions to coronary disease progression when adjusted for treatment status. In this regard, statin and niacin therapy contribute to inter-individual differences LDL-subclass concentrations, enhancing our ability to identify these associations.

The highly significant result for LDL-IIIb would remain significant using Bonferroni correction. Although tables 3 and 4 test multiple associations, the number of primary hypotheses of interest are much more limited. Specifically, the initial report showed that the effects of statin treatment on lesion progression was significant only for those lesions with <30% stenosis at baseline [1]. In addition, prior publications overwhelmingly favor LDL-IIIa, LDL-IIIb, LDL-IVa, and LDL-IVb as being predictive of disease progression [7]. This would suggest only four primary hypotheses, and among these, the reported association between on-study LDL-IIIb and atherosclerosis progression (*P = 10^−8^*, Table 3), and clinical events (P = 0.01, Table 4) would survive Bonferroni adjustment.

Recently, the AIM HIGH (Atherothrombosis Intervention in Metabolic Syndrome with Low HDL/High Triglycerides: Impact on Global Health) trial was terminated for failing to show that raising HDL-cholesterol and lowering triglycerides with niacin treatment resulted in reduced cardiovascular events [19]. The patients, recruited for heart and vascular disease, low HDL-cholesterol, and increased triglycerides, were also administered statin and ezetimibe as required to achieve LDL-cholesterol levels between 40–80 mg/dL. It is possible that when LDL levels are sufficiently low, any cardioprotective effects of raising HDL cholesterol and/or reducing triglycerides with niacin are diminished. The results of the present analyses further suggest that reductions in levels of small LDL particles have a particularly important role in determining the benefits of lipid-lowering therapy on coronary atherosclerosis progression in patients with reduced HDL cholesterol.

Conclusion: In the HATS trial of simvastatin-niacin vs. placebo, low on-study levels of cholesterol in small LDL particles were associated with reduced rate of coronary atherosclerosis progression and the primary clinical cardiovascular endpoint, and these relationships were independent of standard lipid levels. The results support the value of assessing LDL subfractions for the management of cardiovascular disease risk.

## References

[1] Brown BG, Zhao XQ, Chait A, Fisher LD, Cheung MC, et al (2001) Simvastatin and niacin, antioxidant vitamins, or the combination for the prevention of coronary disease. N Engl J Med 345: 1583–1592. 11757504 10.1056/NEJMoa011090

[2] Krauss RM, Burke DJ (1982) Identification of multiple subclasses of plasma low density lipoproteins in normal humans. J Lipid Res 23: 97–104. 7057116

[3] Berneis KK, Krauss RM (2002) Metabolic origins and clinical significance of LDL heterogeneity. J Lipid Res. 43: 1363–1379. 10.1194/jlr.r200004-jlr200 12235168

[4] Krauss RM (2010) Lipoprotein subfractions and cardiovascular disease risk. Curr Opin Lipidol 4: 305–311. 10.1097/MOL.0b013e32833b7756 20531184

[5] Rainwater DL, Mitchell BD, Comuzzie AG, Haffner SM (1999) Relationship of low-density lipoprotein particle size and measures of adiposity. Int J Obes Relat Metab Disord 23: 180–189. 10078854 10.1038/sj.ijo.0800813

[6] Kulkarni KR (2006) Cholesterol profile measurement by vertical auto profile method. Clin Lab Med. 26: 787–802. 10.1016/j.cll.2006.07.004 17110240

[7] Williams PT, Superko HR, Haskell WL, Alderman EL, Blanche PJ, et al (2003) Smallest LDL particles are most strongly related to coronary disease progression in men. Arterioscler Thromb Vasc Biol 2003 23: 314–321. 10.1161/01.atv.0000053385.64132.2d 12588777

[8] Zhao XQ, Kosinski AS, Barnhart HX, Superko HR, King SB (2003) Prediction of native coronary artery disease progression following PTCA or CABG in the Emory Angioplasty versus Surgery Trial. Med Sci Monit. 9: CR48–54. 12601286

[9] Musunuru K, Strong A, Frank-Kamenetsky M, Lee NE, Ahfeldt T, et al (2010) From noncoding variant to phenotype via SORT1 at the 1p13 cholesterol locus. Nature. 466: 714–719. 10.1038/nature09266 PMC3062476 20686566

[10] Strong A, Ding Q, Edmondson AC, Millar JS, Sachs KV, et al (2012) Hepatic sortilin regulates both apolipoprotein B secretion and LDL catabolism. J Clin Invest. 122: 2807–2816. 10.1172/JCI63563 PMC3408750 22751103

[11] Luc G, Fievet C, Arveiler D, Evans AE, Bard JM, et al (1996) Apolipoproteins C-III and E in apoB- and non-apoB-containing lipoproteins in two populations at contrasting risk for myocardial infarction: the ECTIM study. Etude Cas Témoins sur 'Infarctus du Myocarde. J Lipid Res 37: 508–517. 8728314

[12] Sacks FM, Alaupovic P, Moye LA, Cole TG, Sussex B, et al (2000) VLDL, apolipoproteins B, CIII and E and risk of recurrent coronary events in the Cholesterol and Recurrent Events (CARE) trial. Circulation 102: 1886–1892. 11034934 10.1161/01.cir.102.16.1886

[13] Mendivil CO, Rimm EB, Furtado J, Chiuve SE, Sacks FM (2011) Low-density lipoproteins containing apolipoprotein C-III and the risk of coronary heart disease. Circulation 124: 2065–2072. 21986282 10.1161/CIRCULATIONAHA.111.056986 PMC3244212

[14] Shin MJ, Krauss RM (2010) Apolipoprotein CIII bound to apoB-containing lipoproteins is associated with small, dense LDL independent of plasma triglyceride levels in healthy men. Atherosclerosis 211: 337–341. 20303494 10.1016/j.atherosclerosis.2010.02.025

[15] Kawakami A, Aikawa M, Libby P, Alcaide P, Luscinskas FW, et al (2006) Apolipoprotein CIII in apolipoprotein B lipoproteins enhances the adhesion of human monocytic cells to endothelial cells. Circulation 113: 691–700. 16461842 10.1161/CIRCULATIONAHA.105.591743

[16] Hiukka A, Ståhlman M, Pettersson C, Levin M, Adiels M, et al (2009) ApoCIII-enriched LDL in type 2 diabetes displays altered lipid composition, increased susceptibility for sphingomyelinase, and increased binding to biglycan. Diabetes 58: 2018–2026. 19502413 10.2337/db09-0206 PMC2731525

[17] Kawakami A, Yoshida M (2009) Apolipoprotein CIII links dyslipidemia with atherosclerosis. J Atheroscler Thromb 16: 6–11. 19262004 10.5551/jat.e607

[18] Friedewald WT, Levy RI, Fredrickson DS (1972) Estimation of the concentration of low-density lipoprotein cholesterol in plasma, without use of the preparative ultracentrifuge. Clin Chem 18: 499–502. 4337382

[19] AIM-HIGH Investigators (2011) Niacin in Patients with Low HDL Cholesterol Levels Receiving Intensive Statin Therapy. N Engl J Med 365: 2255–2267. 22085343 10.1056/NEJMoa1107579

